# Agnosticism in China

**Published:** 1883-06

**Authors:** 


### AGNOSTICISM IN CHINA.

Every true Confucian, says the *North China Herald*, is an agnostic. He believes only in the seen, the unseen he regards as unknown and unknowable. When asked how we should serve the spirits, Confucius replied, "Unable to serve men, how can we serve spirits?" Confine your thoughts to human duty. To serve men well is the best way to serve the gods. To the question which immediately followed, regarding death, his answer was, "Not knowing life, how can we know death?" Attend to the present; why trouble yourself with insoluble riddles about the future? Life and death are one. Live well and you will die well. Confucius was a thorough-going agnostic. He did not deny the existence of gods and spirits, nor the possibility of a future life. He simply regarded such subjects as beyond human knowledge, and refused to discuss them. He was sure of his five senses, and declined to move a step further. As an agnostic the Confucianist is tolerant of other creeds. He goes even further, and will admit that for the ignorant multitude, and especially for women, an apparatus of gods and demons is necessary. He does not care, therefore, to proclaim his skepticism, still less to actively propagate it. His creed is only for the wise; the masses are better as they are. He will subscribe to the temples, and take part in idolatrous ceremonies. To the common people, Confucian agnosticism has never been very satisfactory. But the agnostic philosophy has not been without its influence on the masses. There is but little religious fervor, and scarcely any deep faith. The people will ridicule their own gods, laugh at their own worship and freely criticise all the creeds. Speak to any Chinese, no matter what his rank, about the future life, and his reply is almost certain to be, "Who knows anything about it?" and is likely enough to add, "Eating and drinking are realities," implying that all else is doubtful. Refer to the subject of future rewards and punishments, and his sarcastic remark will probably be, "I have seen the living suffer, but never seen the dead in cangues." The present is certain; the future is all unknown. He therefore keeps a sharp eye to the present chance. It must be now or never; there may be no to-morrow. Intense worldliness and general animalism are the natural results. The conclusion of the whole matter shows how far superior morally the original and orthodox systems of Buddhism and Taoism are to the agnostic attitude.

---

**A GIRAFFE has never been known to utter a sound. In this respect it resembles a young lady in a street car when a gentleman gives her his seat.**

